# Pulmonary adenoid cystic carcinoma ‐ a spectrum of disease: two case reports

**DOI:** 10.1002/rcr2.1230

**Published:** 2024-02-08

**Authors:** Margaret Gleeson, Megan Finan, Karen Redmond

**Affiliations:** ^1^ Respiratory Mater Misericordae University Hospital Dublin Ireland

**Keywords:** bronchoscopy and interventional techniques, histology, lung cancer, rare lung disease, thoracic surgery

## Abstract

Adenoid cystic carcinoma (ACC) is a rare form of adenocarcinoma that usually begins in the oral cavity, with most cases arising from the salivary glands. Owing to its low incidence, the precise clinical and pathological features, including therapeutic strategy and survival data have not been conclusively reported. ACCs are typically characterized by slow growth, perineural invasion with local and often late recurrence after initial diagnosis. However, some cases demonstrate unusual aggressive biologic behaviour. Herein we describe our experience of two patients with a diagnosis of ACC. These cases highlight the spectrum of the disease with individualized treatment strategies.

## Graphical abstract

Although encountered infrequently in clinical practice, pulmonary adenoid cystic carcinoma is typically a slow‐growing indolent condition however, a small proportion of patients present with acute, progressive symptoms and represent a diagnostic and therapeutic challenge. Novel treatments including proton beam therapy and 3D stent reconstruction have been successfully used in this cohort.

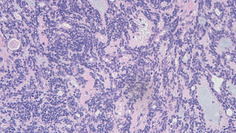

## INTRODUCTION

Adenoid cystic carcinoma (ACC) is a malignant neoplasm that most frequently originates from the major and minor salivary glands of the head and neck. It uncommonly arises from other tissues with nose, lung, breast, skin, upper GI tract, cervix and prostate described in the literature. Primary pulmonary adenoid cystic carcinoma (PACC) is rare, being responsible for 17% of all ACCs, and accounting for <0.2% of all primary lung malignancies. Due to its rarity, the natural history of this tumour is not well defined and no current guidelines exist to guide diagnosis or treatment.

## CASE REPORT

### Case 1

**FIGURE 1:**
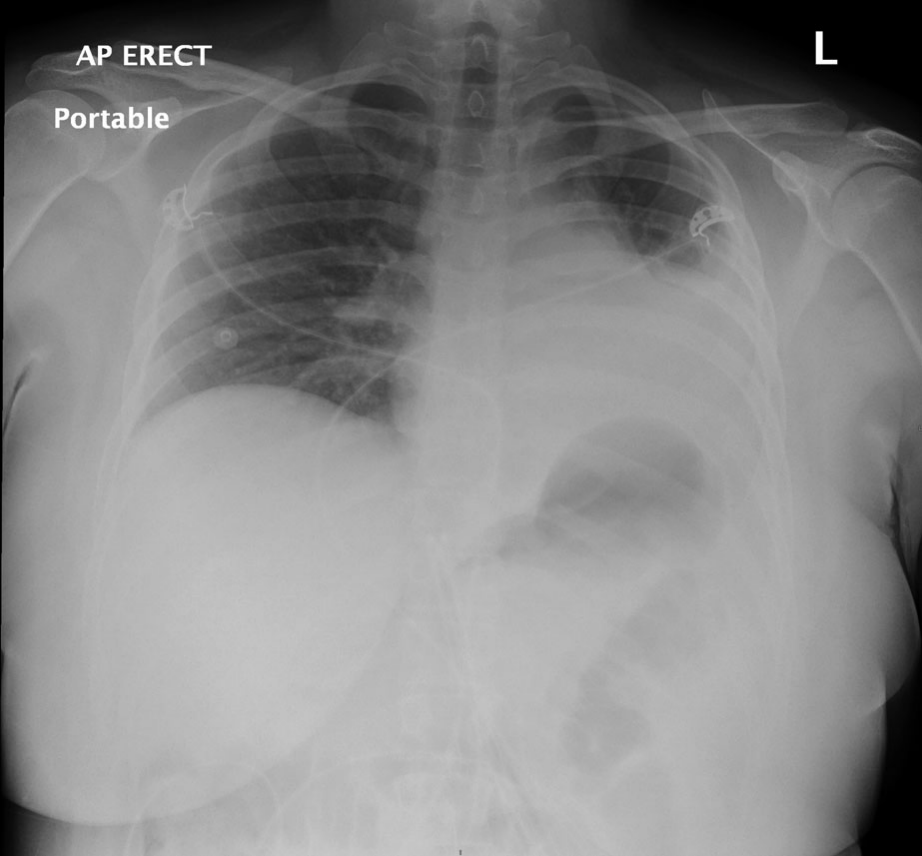
Left lower lobe collapse.

A 62‐year‐old female with a remote smoking history presented with a more gradual history with symptoms of a lower respiratory tract infection including wheeze, dyspnoea and cough. A relapse in symptoms approximately 2 months later following treatment resulted in a referral to the hospital prompting imaging. CT thorax revealed a lesion above the carina narrowing the airway lumen and biopsy confirmed a PACC, with a cribiform pattern. Multidisciplinary discussion determined her disease as operable. Positive histological margins required adjuvant radiation remaining disease‐free at follow‐up (Figure 1).

### Case 2

**FIGURE 2:**
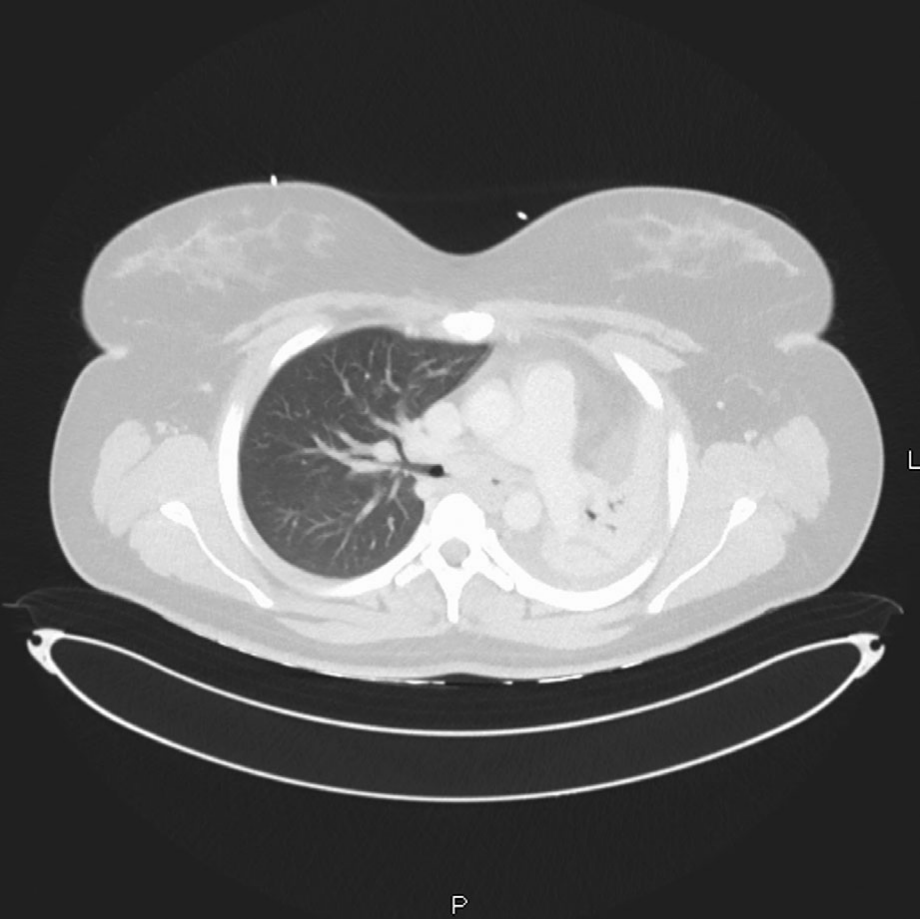
Left main bronchus mass with left lung collapse.

**FIGURE 3:**
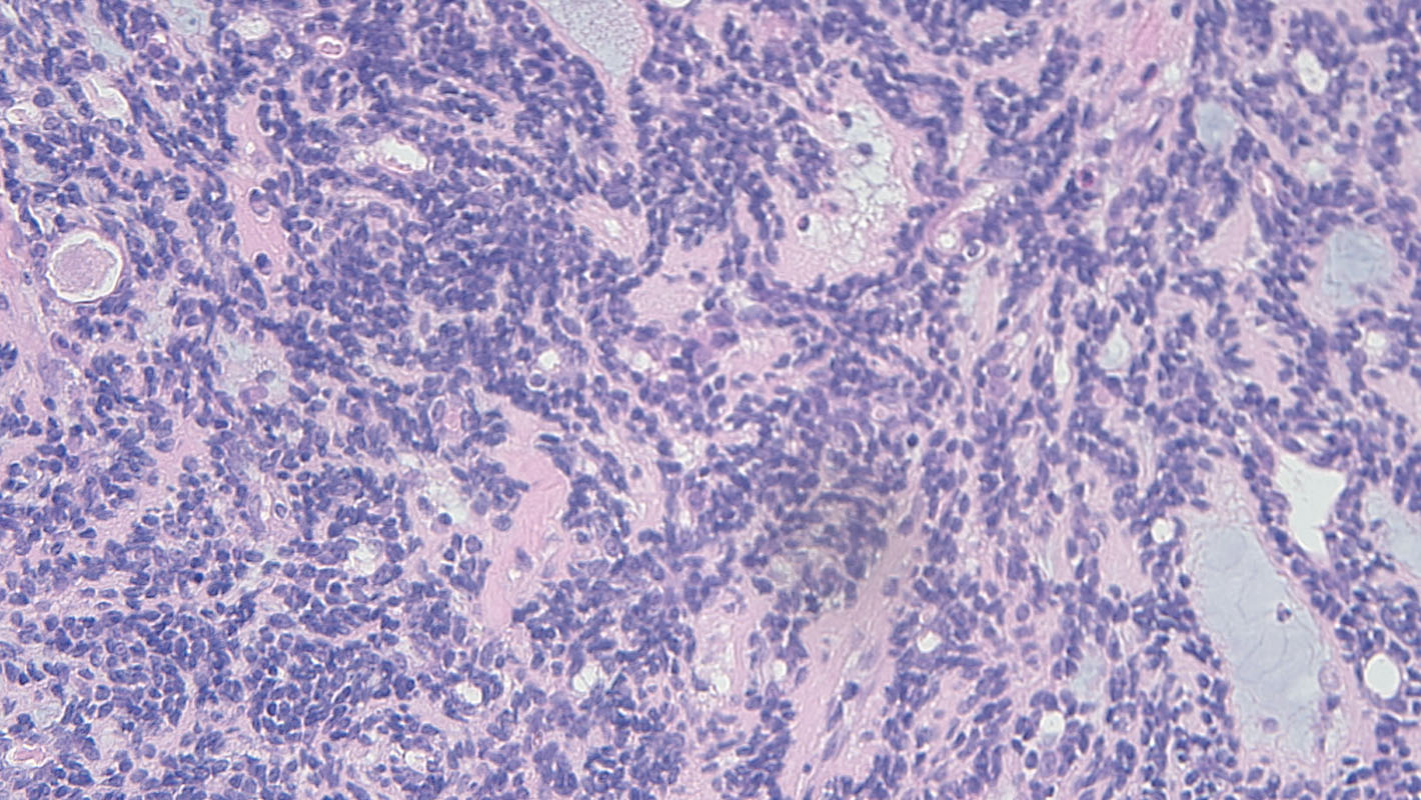
Tracheal biopsy showed extensive infiltration of the respiratory mucosa by a malignant epithelial neoplasm. Tumour nests of basaloid cells are seen in the solid pattern of adenoid cystic carcinoma (ACC). The solid pattern tends to be more mitotically active and has a poorer prognosis than other types of ACC.

A 22‐year‐old female presented to the Emergency Department with an acute history of shortness of breath and chest pain. She was a lifelong non‐smoker. Significant symptom burden and concern for airway compromise led to a CT thorax which revealed a mass at the level of the left main bronchus with mediastinal shift and left lung collapse (Figures 1 and 2). A flexible bronchoscopy confirmed an endobronchial lesion the left main bronchus extending towards the carina with subsequent rigid bronchoscopy facilitating debridement to maintain airway patency as well as tissue sampling. ACC was confirmed with a solid variant on histological examination (Figure 3). In addition to endoscopic intervention she was referred for proton beam therapy and at follow up was symptom‐free.

## DISCUSSION

ACCs in general have a widespread age distribution and usually present with a good performance status at presentation. There is often a significant delay between symptoms onset and accurate diagnosis, averaging between 16 and 24 months^1^, ^2^ This is in part due to its rarity and clinician unfamiliarity but also the younger age distribution leading to misdiagnosis of more commonly seen pathologies such as asthma, bronchitis or a lower respiratory tract infection.^3^

When the lower respiratory tract is affected it originates from the tracheobronchial glands distributed in the airway submucosa. It is more commonly found in the central airways, classically the upper third of the trachea, but extends into the main bronchi rather than segmental bronchi in over 90% of cases.^4^

As reflected in our case series ACC can have a highly variable clinical course.^5^ As in our first case it typically manifests as an indolent and slow‐growing tumour with mild symptoms followed by repeated or escalating presentations to healthcare settings. Pulmonary ACCs may grow to remarkable sizes or demonstrate significant local invasion before presentation. More rarely, as in case two, it grows aggressively with sudden onset of severe or even life‐threatening symptoms with rapid and refractory progression of obstructive symptoms with dyspnoea, cough, wheeze and stridor described.^6^

**FIGURE 4:**
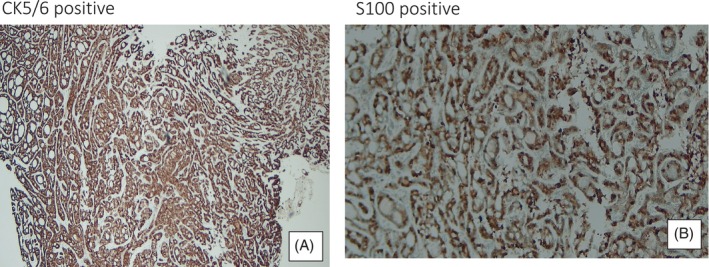
In order to rule out other lesions with histopathological characteristics similar to adenoid cystic carcinoma immunohistochemistry profiling was performed. CK 5/6 and s100 were positive as seen above demonstrating presence of myoepithelial cells. A/E 13 and CD 117 were also positive—a profile which is supportive of the diagnosis. Chromagranin and synaptophysin, neuroendocrine markers, were negative.

Histology is confirmatory and PACC are characterized by three distinctive architectural patterns, the most frequent being ‘cribiform’, followed by a tubular form.^7^ These exhibit low grade disease and have a long‐term good prognosis. The less common solid variant is the most aggressive demonstrating increased mitotic activity and subsequently present with more aggressive disease and pare associated poorer prognosis as in case one (Figure 4).^8^

Imaging is important for guiding tissue sampling and determining location, size and spread of the tumour. ACCs are known for their regional metastatic spread and local invasion, particularly infiltration along the neural lining of the tracheal wall.^9^ Imaging can select patient that are candidates for surgical resection and can identify those at risk of poor pathological clearance. CT is the most common modality but with increasing interest is PET‐CT, with primary PACCs and distant metastases demonstrating intense FDG activity. This translates to high sensitivity in those post treatment looking for residual or recurrent disease on surveillance imaging.^10^

Due to its rarity and the lack of prospective data, no guidelines have been established on the management of PACCs and information is largely extrapolated from institutional experiences and published case reports.

Definitive surgical management is considered first line treatment with superior outcomes and curative intent.^11^ In our first case, resection to a level compatible with a good outcome was deemed likely and tracheal resection with reconstruction was performed. Cases may be considered inoperable when patients are deemed medically unfit for surgery due to age or comorbidities or when the disease is advanced or disseminated. Unresectable disease describes cases where excision with suitable reconstruction is unlikely as in our first case. For these patients, endoscopic palliation including coring, diathermy, cryo‐, argon beam or laser therapy can be performed.^12^, ^13^ Even for those with advanced or metastatic disease, survival may be years and here the goal is to improve and prolong symptom‐free survival. This approach can have comparable outcomes to radical resection even when the risk of recurrent or residual disease is considered. While these procedures may avoid the morbidity associated with extensive resection, the main disadvantage of this approach is symptom recurrence and the need for repeated procedures as in the case of second our patient to maintain airway patency. Furthermore, repeated airway debulking can be complicated by trachobronchomalacia which can itself result in symptoms or reduced quality of life. More recently, novel patient specific 3D printed airway stents have been developed to help maintain airway patency due to this, disease recurrence or as a bridge to therapy, and this to our knowledge is the first described in a patient with tracheomalacia secondary to PACC.^14^

ACCs are for the most part chemo‐resistant with no sustained treatment benefit reported in this cohort.^15^ The role of targeted therapies such as TKI and immunotherapy remains unclear and requires further research.

Radiation sensitivity is high and can be a first line definitive treatment for inoperable cases, particularly those with significant symptom burden such as our patient^16^ or adjuvant therapy in up to 70% of post‐operative cases.^11^

It can also be used adjunctively for patients with incomplete resection, with surgical microscopic or grossly positive margins, where the risk of recurrence is high and where the aim is to target residual disease. Those with perineural invasion or lymph node spread may also benefit from improved early local control. Be it definitive or adjuvant treatment, owing to its anatomical proximity to significant organs it can be associated with significant toxicity. There is a role for both endo‐luminal and external beam radiotherapy, with complete remission reported at 64 months of follow‐up, for patients with unresectable ACC involving the trachea, carina and bronchi More recently proton beam therapy, as a form of external beam radiation, has become an option.^17^ This is advantageous in cases where the anatomical location is difficult and the risk of toxicity high. It has been associated with excellent early local disease control and also low rates of early and late complications. The maximal dose deposition and little exit radiation to by standing organs, notably the surrounding lung, heart and oesophagus.^18^ Case number one is consistent with published case series demonstrating good outcomes with this treatment approach. Importantly the reduced risk of secondary malignancies associated with proton therapy is relevant given the young age and long term survival of many of these patients.^19^

In conclusion, despite a historic slow growing nature, our case series reflects the anecdotal spectrum experience in real world clinical practice. This tumour demonstrates huge variability not only in histopathology but also the acuity of presentation, symptomatology and prognosis. The unfamiliarity and unpredictability of its varied clinical course and lack of current evidence‐based guidelines suggest a specialist centre is most appropriate for managing such patients.

## AUTHOR CONTRIBUTIONS

**Margaret Gleeson**: Author. **Megan Finan**: Pathologist; author. **Karen Redmond**: Cardiothoracics; author; supervisor.

## CONFLICT OF INTEREST STATEMENT

None declared.

## ETHICS STATEMENT

The authors declare that appropriate written informed consent was obtained for the publication of this manuscript and accompanying images.

## Data Availability

The data that support the findings of this study are available from the corresponding author upon reasonable request.
